# Supplementary material for: Demonstration of tritium adsorption on graphene
Source: Nanoscale Adv. 2024 Mar 27;6(11):2838–49. doi: 10.1039/d3na00904a (PMC11134268; doi:10.1039/d3na00904a)
Supplement: NA-006-D3NA00904A-s001 [file NA-006-D3NA00904A-s001.pdf]

Electronic Supplementary Material (ESI) for Nanoscale Advances.
This journal is © The Royal Society of Chemistry 2024

Supplementary Information

Demonstration of tritium adsorption on graphene

Genrich Zeller,*[a] Desedea Diaz Barrero,[a,b] Paul Wiesen,[a] Simon Niemes,[a] Nancy Tuchscherer,[a] Max Aker,[a] Artus M. W. Leonhardt,[a] Jannik Demand,[a] Kathrin Valerius,[c] Beate Bornschein,[a] Magnus Schlösser [a] and Helmut H. Telle [b]

[a] *Tritium Laboratory Karlsruhe (TLK), Institute for Astroparticle Physics (IAP), Karlsruhe Institute of Technology (KIT), Hermann-von-Helmholtz-Platz 1, 76344 Eggenstein-Leopoldshafen, Germany*

[b] *Departamento de Química Física Aplicada, Universidad Autónoma de Madrid, Campus de Cantoblanco, 28049 Madrid, Spain*

[c] *Institute for Astroparticle Physics (IAP), Karlsruhe Institute of Technology (KIT), Hermann-von-Helmholtz-Platz 1, 76344 Eggenstein-Leopoldshafen, Germany*

[*] Corresponding author; eMail – genrich.zeller@kit.eu

Supplement S1 – Measurement protocol

The overall measurement methodology can be separated into two main action blocks, i.e., (i) the tritiation process of the graphene samples; and (ii) the T-graphene characterisation after termination of the exposure (see Fig. 2 in Section 2.4 of the main publication text).

The full sequence of the preparation / characterisation protocol is as following.

(1) The four graphene samples are placed in a custom-made loading chamber (samples A to D, see Fig. S1), and the central sample (A) is electrically contacted for *in situ* sheet resistance measurement. During the whole sample-preparation procedure – steps 2 through to 7 – temperature and sheet resistance are continuously monitored.

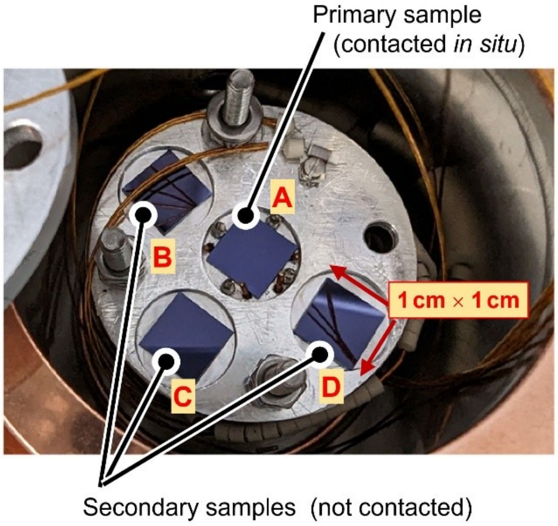

Fig. S1: Sample holder within the tritium loading-chamber, with four mounted graphene samples: sample A – electrically contacted; samples B to D – without electrical contacts. For the use of the different samples, see text.

Sample preparation (tritiation)

(2) The chamber is evacuated for several hours, to remove as many of the potential molecular contaminants as possible.

(3) Several heat-cycles are performed to determine the temperature coefficient of resistance of sample A prior to tritium exposure (see Fig. S2(A) of this Supplement).

(4) The samples are exposed to tritium for 55 h (at about 400 mbar).

(5) After exposure, the chamber is evacuated for several hours, to remove any physisorbed tritium from the samples and the stainless-steel walls of the chamber.

(6) The samples are heat-cycled several times between ~25 °C and ~110 °C, to determine whether the temperature coefficient of resistance has changed due to the exposure to tritium (see Fig. S2(B) of this supplement).

(7) Finally, the loading chamber is evacuated for ~7 days to substantially less than 10^{-3} mbar, to remove any tritium still out-gassing from the stainless-steel walls of the chamber. This is required to keep any tritium contamination in the glovebox at very low levels when opening the chamber for removal of the samples; the time span for sample removal should be as short as possible.

Sample characterisation

(8) The removed samples are stored within the glovebox, prior to the characterisation measurements.

(9) Sample (B) is heated in the oven for 5h at 1400 °C, and the total activity released from the graphene sample is determined; of course, at this high temperature tritium is also liberated from the SiO_2/Si substrate bulk. In contrast to the heating sequence outlined in step (11) below, this procedure is destructive; most of the graphene film vanishes.

(10) For the non-contacted sample (C) Raman spectra are recorded, using the confocal Raman microscope, for a selected area at the centre of the sample. The related Raman spectra and maps are used for comparison in the Raman measurements of the contacted sample (A).

(11) For the contacted sample (A) Raman spectra are recorded, using the confocal Raman microscope. First, the full area of the sample is scanned with spatio-spectral resolution. Then a smaller, selected area at the centre of the tritiated sample is scanned (to limit the measurement time). The overall sequence encompasses the following steps:
    a. initial Raman scan in a central area of the sample;
    b. the oven is flushed with “wet” Argon gas (flowing through a water wash bottle the gas saturates with water vapor) to prevent oxidation of the graphene layer during heating;
    c. a first repeat Raman scan is performed after the 1st heating of the sample (for 3.5h at to 300 °C);
    d. a second repeat scan is performed after the 2nd heating (for 21h at 300 °C); and
    e. a third and final repeat scan is carried out after the 3rd heating (for 22h at 500 °C).

    After each step, the individual, released activity is measured.

(12) Sample (D) is stored under dry N_2 gas for later out-gassing and stability measurements over extended time; note that these had not yet been completed at the time of writing.

Supplement S2 – *In situ* monitoring during sample heating

In situ sample heating

We found that the van der Pauw measurement was failing after vacuum-heating of graphene at temperatures above 120 °C, probably related to thermal stress in contact between graphene and the spring-loaded electrodes. Thus, we limited the maximum temperature to 110 °C. Before the samples are heated the chamber is evacuated for 10 h, or until the pressure inside the chamber reaches ~10^{-6} mbar. During heating the vacuum pumps remain running; therefore, all released adsorbates are promptly removed from the system.

The heating is conducted slowly to ensure that the heater, the samples, and the temperature sensor are in thermal equilibrium. It takes ~2 h for the sample temperature to reach 110 °C when starting at room temperature. The cooling of the samples is completely passive and can take 6-8 h to return to room temperature.

Temperature dependence of resistance

Using the internal heating element, the temperature dependence of the sheet resistance of graphene samples was studied. These measurements were conducted before and after tritium exposure. Before the graphene sample was exposed to tritium its sheet resistance increased, as a function of temperature, with a Temperature Coefficient of Resistance (TCR) of $TCR_{pre} = 1.13\times10^{-3}$. This behaviour and its order of magnitude are comparable to previous studies on pristine graphene samples.[66] Fig. S2(A) shows the measurement data in the form $\ln R$ vs T^{-1}. With a linear fit to the data, we derive band gaps in the order of –10 meV. After tritium exposure the opposite correlation between R and T is observed ($TCR_{post} = -3.56\times10^{-3}$), with a bandgap of 40 meV, as shown in Fig. S2(B).

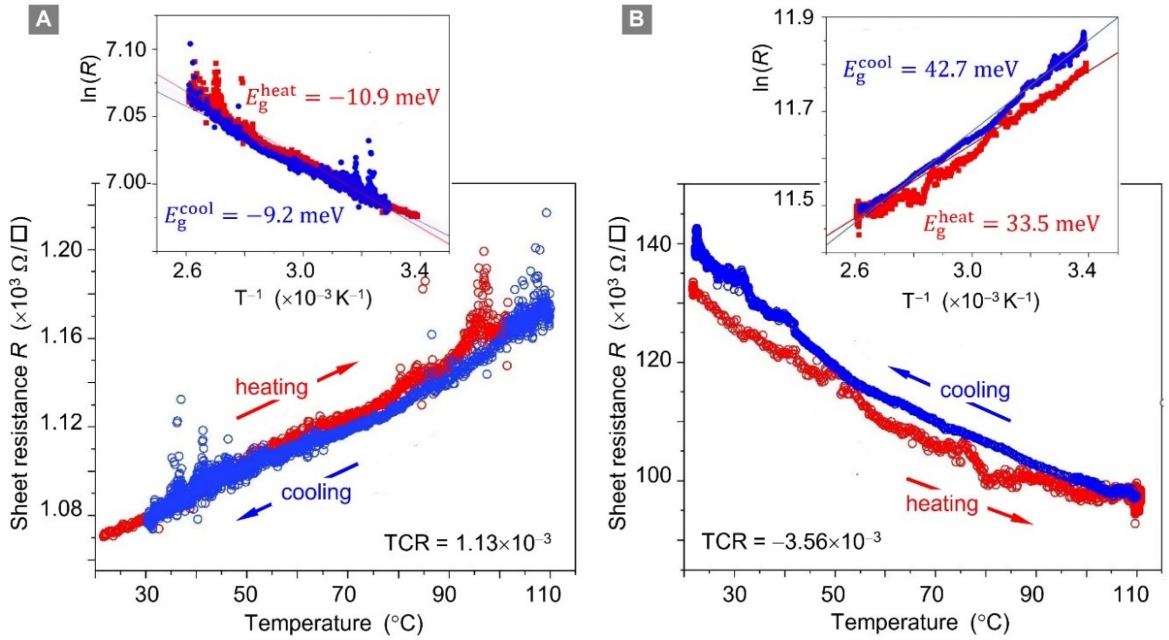

Fig. S2: Temperature dependence of the sheet resistance R(T) before (A) and after tritium exposure (B); the values for the slope (TCR) are indicated. Inserts: logarithmic presentation of the sheet resistance, with $\ln(R) \sim \exp(E_g/k_BT)$; values E_g for during sample heating and cooling are indicated (note the different slopes).

Reference

66 Lei, S., Su, N. and Li, M.; Thermal-resistance effect of graphene at high temperatures in nanoelectromechanical temperature sensors, *Micromachines*, 2022, **13**, 2078. https://doi.org/10.3390/mi13122078

Supplement S3 – Setup of the confocal Raman microscope

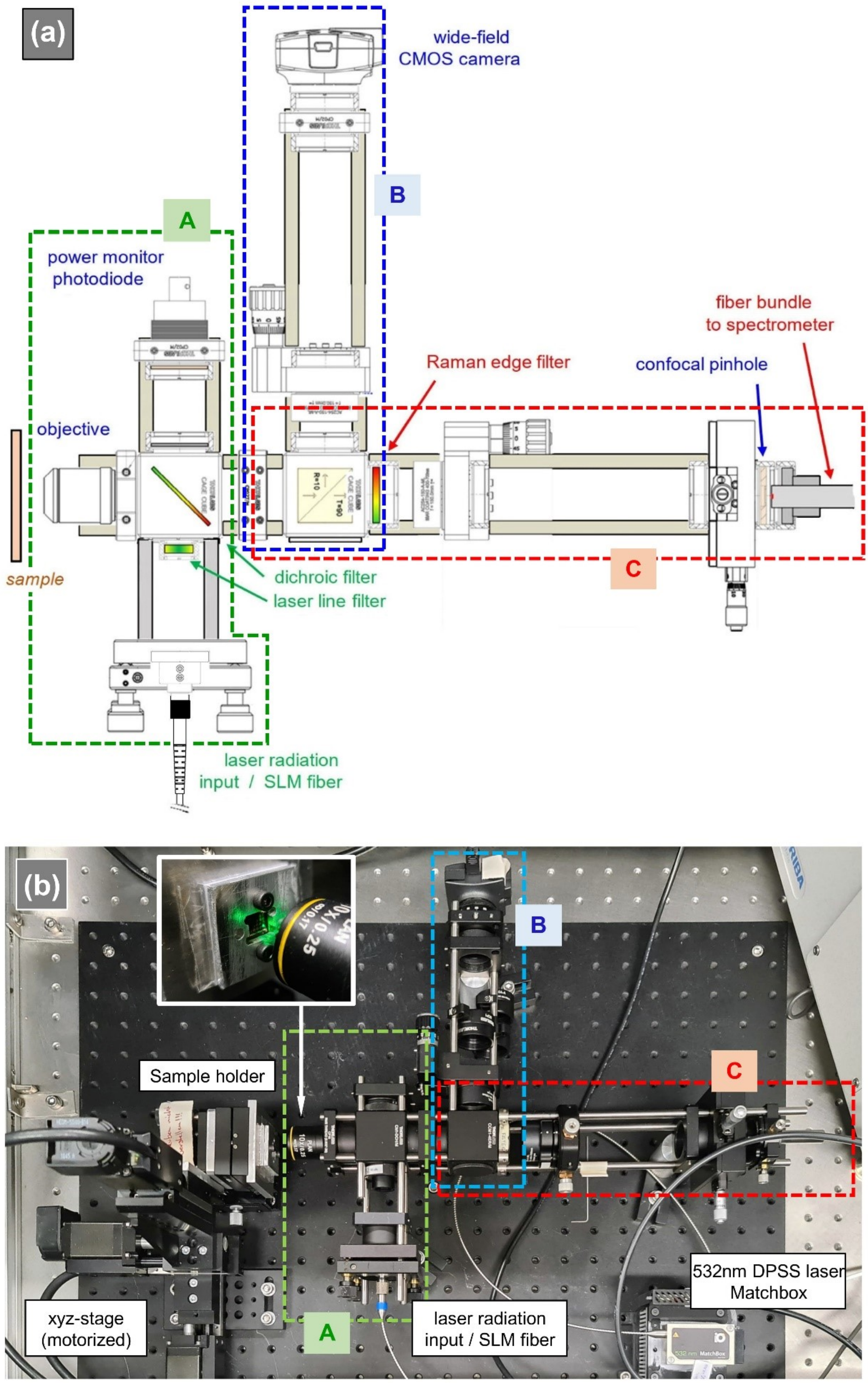

Fig. S3: Confocal Raman microscope (CRM); (a) conceptual sketch; (b) photo of the actual system used in this work. Note: the tritium protection shield between the microscope objective and the sample holder is not installed here, for clarity. For details see D. Diaz Barrero *et al*, *Sensors*, 2022, **22**, 10013.

Supplement S4 – Raman maps of pristine graphene

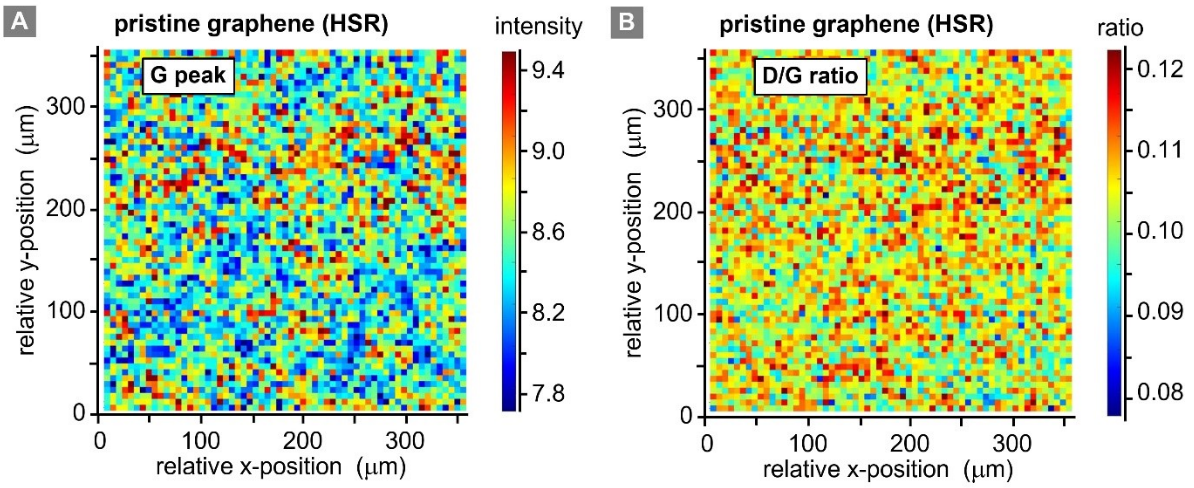

Fig. S4: Raman raster scans a pristine graphene-on-SiO_2/Si sample (*Graphenea*). (A) – Raman map of the graphene G-peak signal, for a 350×350 μm^2 sample section pre-tritium exposure; step size ΔS=5 µm. (B) – graphene D/G Raman peak-ratio map, pre-tritium exposure.
